# Randomized controlled trial of pegbovigrastim as an adjunct therapy for naturally occurring severe clinical mastitis cases in dairy cows

**DOI:** 10.3168/jdsc.2021-0137

**Published:** 2021-08-20

**Authors:** J. Denis-Robichaud, M. Christophe, J.-P. Roy, S. Buczinski, M. Rousseau, M. Villettaz Robichaud, J. Dubuc

**Affiliations:** 1 Independent researcher, Amqui, Québec, Canada, G5J 2N5; 2 Département de sciences cliniques, Faculté de médecine vétérinaire, Université de Montréal, 3200, rue Sicotte, St-Hyacinthe, QC, Canada, J2S 2M2

## Abstract

The main objective of this study was to quantify the effect of pegbovigrastim (PEG) as an adjunct therapy for naturally occurring severe mastitis cases on survival, intramammary bacteriological cure, and subsequent milk production. A double-blinded, randomized controlled trial was conducted on a single commercial dairy farm. During the study period, all cows having a case of severe clinical mastitis, defined as the presence of abnormal milk and inflammation in one quarter or more combined with the presence of systemic signs (pyrexia, dehydration, or recumbency), were enrolled in the study. In addition to a standardized therapy combining systemic and intramammary antimicrobials as well as systemic anti-inflammatory drugs, cows received a subcutaneous injection of either 2.7 mL of 0.9% sterile saline (control group; CON) or 2.7 mL of PEG (PEG group). A milk sample for bacteriological analysis was taken before treatment was administered and a second sample was taken 14 d after enrollment. Survival (30 d post-treatment) and bacteriological cure (14 d post-treatment) were analyzed by survival analysis and chi-squared tests, respectively, whereas daily milk production was treated as a repeated measure in mixed regression models. The 77 cows enrolled in the study were of parity 2 to 4 (mean = 3) and between 3 and 302 d in milk (mean = 154). The bacteria identified in milk at enrollment were *Klebsiella* spp. (n = 48; 62%), *Escherichia coli* (n = 16; 21%), *Enterobacter* spp. (n = 10; 13%), or no growth (n = 3; 4%). The probability of survival during the first 30 d after treatment was higher in the PEG group (84%) than in the control group (46%). Daily milk production over the 30-d period following treatment and bacteriological cure 14 d post-treatment did not differ between groups. Overall, cows treated with PEG as an adjunct therapy for naturally occurring cases of severe clinical mastitis had a better survival than cows treated with saline.

## Highlights

• Treatment with pegbovigrastim increased survival after a case of severe clinical mastitis.
• Treatment with pegbovigrastim had no effect on subsequent bacteriological cure.
• Treatment with pegbovigrastim had no effect on subsequent milk production.

## Graphical Abstract

**Figure:**
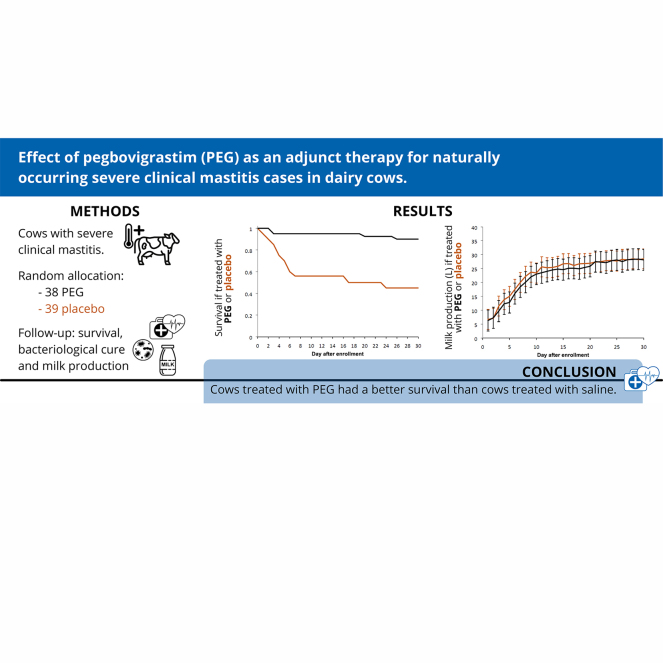
**Summary:** The main objective of this controlled randomized trial was to quantify the impact of pegbovigrastim as an adjunct therapy for severe clinical mastitis cases in dairy cows. A total of 77 cows were enrolled in the study. Cows treated with pegbovigrastim had an improved survival (30 d after treatment) compared with cows that received saline (control), but the treatment had no effect on bacteriological cure at 14 d post-treatment or on daily milk production during the 30-d period following enrollment. These findings improve our understanding of the effect of pegbovigrastim as an adjunct treatment on survival and health in severe mastitis cases in cows.

Intramammary infections are a major economic burden for dairy producers. Clinical mastitis events result in direct and indirect costs due mainly to increased culling, milk yield reduction, milk discard, and treatment (Rollin et al., 2015; Aghamohammadi et al., 2018). Severe clinical mastitis, defined as abnormal milk, abnormal udder, and systemic signs of illness, also takes its toll on dairy farms, with up to 15% of cases resulting in death of the affected cow (Erskine et al., 2002; Pinzón-Sánchez and Ruegg, 2011; Ruegg, 2012).

Pegbovigrastim (**PEG**) is the polyethylene glycolated form (covalently bound) of bovine granulocyte colony-stimulating factor, a growth factor targeting hematopoietic progenitor cells, stimulating production and differentiation of neutrophils, and it was developed to improve immunity of dairy cows during the transition period (Hassfurther et al., 2015). In Canada, PEG (Imrestor, Elanco) is labeled for the reduction in incidence of clinical mastitis in the first 30 d of lactation in periparturient dairy cows and replacement heifers. In this context, 2 doses of PEG need to be administered: the first 1 wk before the expected due date and the second within 24 h after calving. Recent studies showed that such use of PEG increased circulating neutrophils around parturition (Kimura et al., 2014; van Schyndel et al., 2018). It was also associated with decreased odds of clinical mastitis during the first 30 d of lactation compared with untreated cows (Hassfurther et al., 2015; Canning et al., 2017; Ruiz et al., 2017), but there was no diminution in the odds of severe mastitis, perhaps due to the low incidence observed in the study (0.2%; Ruiz et al., 2017). As stated on the Canadian label, the current use of PEG is preventative. Although prevention is of utmost importance, cases of clinical mastitis are still happening on dairy farms, with a reported herd incidence rate varying widely, from 1 to 97 cases per 100 cow-years (Olde Riekerink et al., 2008). Furthermore, 5 to 14% of clinical mastitis cases have been reported to be severe (Erskine et al., 2002; Pinzón-Sánchez and Ruegg, 2011; Ruegg, 2012).

During a clinical mastitis case, PMN are recruited rapidly in the mammary gland; they represent the most effective defense against bacterial infection when they ingest pathogens by phagocytosis (Paape et al., 2002). While multiple biological elements are in play to control IMI and inflammation, increased recruitment of neutrophils could mitigate mortality and improve the bacteriological cure and subsequent milk production of affected cows, especially in severe mastitis cases. Thus, the use of PEG as an adjunct therapy in these cases could improve the survival and performance of cows.

The main objective of this study was to quantify the effect of PEG as an adjunct therapy on survival, intramammary bacteriological cure, and subsequent milk production in cows with naturally occurring severe mastitis. The hypothesis was that PEG would improve survival in the 30 d after treatment, bacteriological cure 14 d post-treatment, and milk production in the 30 d following treatment.

This double-blinded randomized controlled trial (cow level) was approved by the Animal Care Committee of the Université de Montréal (#18-Rech-1985), and the REFLECT statement was used to report the findings (O'Connor et al., 2010). The trial was conducted between July 2018 and April 2021 in a single herd of 300 lactating Holstein cows (QC, Canada) in which cows were housed in a freestall facility and fed a TMR. This herd was selected by convenience based on being located within 1 h of the bovine ambulatory clinic of the Faculté de médecine vétérinaire of the Université de Montréal (St-Hyacinthe, QC, Canada), on having a high incidence of naturally occurring cases of severe clinical mastitis [lactational incidence risk of 11.3% between June 2017 and June 2018; 94% (gram-negative bacteria) and 6% (no growth) were identified using the on-farm Tri-Plate culture system (University of Minnesota) during that period], for not already using PEG on the farm, and for being willing to participate in the present study. Severe clinical mastitis was defined as the presence of abnormal milk and inflammation in one quarter or more, combined with systemic signs of illness (pyrexia, dehydration, or recumbency; Pinzón-Sánchez and Ruegg, 2011). This definition was standardized for farm staff before and during data collection. The farm manager was in charge of the identification, sampling, and treatment of cases of severe clinical mastitis.

A sample size of 77 cows (35 cows per treatment group + 10% loss to follow-up) was targeted for this study based on 3 different sample size estimations following results of a pilot study. The first (20 cows per treatment group) was based on finding a difference in the proportion of cows surviving the first week following the mastitis case (50% in the control group vs. 90% in the PEG group), with 95% confidence and 80% power (Dohoo et al., 2009). The second (20 cows per treatment group) was based on finding a difference in the proportion of cows having bacteriological cure 14 d after enrollment (10% in the control group vs. 50% in the PEG group), with 95% confidence and 80% power. The third (23 cows per treatment group) was based on finding a significant difference in milk production of 5 kg/d (25 kg in the control group and 30 kg in the PEG group) considering a variance of 36 with 95% confidence and 80% power.

When a case of severe clinical mastitis was diagnosed on the farm, the cow was enrolled in the study. At enrollment, all cows were assigned randomly to 1 of 2 treatment groups: (1) control group (**CON**; subcutaneous injection of 2.7 mL of 0.9% sterile saline) or (2) PEG group (subcutaneous injection of 2.7 mL of PEG; Imrestor, Elanco). The syringes were numbered (1 to 77) before the start of the study and assigned randomly using a random number generator (Excel spreadsheet; Microsoft Corp.) to contain saline or PEG (balanced within groups of 10 syringes); this procedure was carried out by a technician not involved in farm data collection. The syringes from both groups were identical and could not be differentiated on the farm. Farmers were blinded to treatment allocation. The syringes were refrigerated at 4°C until use. At enrollment, rectal body temperature was taken using a thermometer. A milk sample from the affected quarter was taken aseptically by the herd manager from all cows. All samples were kept on ice until arrival at the bovine ambulatory clinic (within 6 h).

Once milk was taken, cows were administered a subcutaneous injection in the neck using the prepared syringes (saline or PEG) following the preassigned numerical order (1 to 77). At the same time, all cows received a standardized therapy made of 16 mg/kg trimethoprim-sulfamide IM (Borgal; Merck Animal Health) twice daily for 5 d, 0.5 mg/kg of meloxicam IV (Metacam; Boehringer Ingelheim) once at enrollment, intramammary ceftiofur (Spectramast LC; Zoetis) in the affected quarter once daily for 2 d, and 2 L of homemade 7.5% hypertonic saline solution IV once at enrollment. Ad libitum fresh water was offered to all treated cows after receiving the hypertonic saline solution.

On d 14, a second milk sample from the affected quarter was collected aseptically from all cows. Parity and DIM were obtained from farm records, and culling or death dates, as well as the daily milk production of all enrolled cows, were recorded for 30 d by farm staff (from milking parlor data; DairyPlan C21).

All milk samples were aseptically collected after the teat end was swabbed with gauze soaked in 70% methanol, immediately placed on ice, and sent to the veterinary diagnostic laboratory of the Université de Montréal (St-Hyacinthe, QC, Canada) for standard bacteriological analysis. Briefly, a volume of 10 µL from the milk sample was cultured on Columbia blood agar (5% sheep blood) and the remaining sample was incubated. Cultures were read after 16 h of incubation; if negative, the incubated milk sample was plated on Columbia blood agar and the primary cultures re-incubated for 24 h. Milk samples with growth of ≥3 bacterial species were considered contaminated. Cultures with ≥1 cfu at 48 h were considered positive. Colonies were counted (cfu/mL) and identified using MALDI-TOF MS technology. One colony from each bacterial species was transferred to the MALDI target plate (Bruker Daltonics), overlaid with 0.7 µL of 70% formic acid, and allowed to dry. One microliter of matrix solution (α-cyano-4-hydroxycinnamic acid in 50% acetonitrile and 2.5% trifluoroacetic acid) was then applied to each spot and allowed to dry. Plates were run on a Microflex LT/SH mass spectrometer (Bruker Daltonics). Generated spectra were compared with spectra from the RUO Biotyper database (Bruker Daltonics) and assigned a score based on similarity. When the primary culture yielded no bacterial growth even after 48 h of incubation, the culture from the incubated milk sample was read. Bacteriological cure was defined as the absence 14 d later of the bacteria identified at enrollment.

Statistical analyses were conducted using R version 4.0.0 (R Core Team, 2015). Median, mean, minimum, and maximum were used to describe continuous data, and proportions were used to describe categorical data. Cows were categorized according to their lactation stage (early: <75 DIM, mid: 75–174 DIM, and late: ≥175 DIM). Parity, lactation stage, DIM, rectal temperature, and identified bacteria in milk at enrollment were compared between cows from the control and PEG groups using a Wilcoxon rank sum test for continuous data and a chi-squared test for categorical data. The treatment's associations with bacteriological cure at d 14 were assessed using a chi-squared test (stats package; R Core Team, 2015). The association between treatment and daily milk production during the 30 d following the mastitis event was assessed using Satterthwaite's method ANOVA for repeated measures in a mixed linear regression model (lmer; lme4 package in R), adjusting for DIM as a fixed effect and for the cow as a random effect). Death or culling in the month following treatment was assessed using the log-rank test and the Kaplan-Meier estimator (survival package in R). Dead or culled cows were treated as loss to follow-up and were not included in analysis after their removal from the herd. Statistical significance was considered if *P* ≤ 0.05.

A total of 77 cows were enrolled in the study (39 in CON; 38 in PEG). No adverse events were noted during the study. Cows enrolled in the study were of parity 2 to 4 (median = 3; mean = 3) and between 3 and 302 DIM (median = 148; mean = 154) at enrollment. Nineteen cows were in early lactation (10 in CON: 3 to 62 DIM; 9 in PEG: 15 to 50 DIM), 27 were in mid lactation (12 in CON: 125 to 154 DIM; 15 in PEG: 81 to 171 DIM), and 31 were in late lactation (17 in CON: 189 to 257 DIM; 14 in PEG: 201 to 286 DIM). Their body temperature at enrollment was between 38.0 and 41.0°C (median = 39.7°C), and the bacteria identified in milk at enrollment were *Klebsiella* spp. (n = 48; 62%), *Escherichia coli* (n = 16; 21%), *Enterobacter* spp. (n = 10; 13%) or no growth (n = 3; 4%). At d 14, a total of 25 cows (PEG: n = 13; CON: n = 12) were found with bacteria in their milk, which were identified as *Klebsiella* spp. (n = 22; 88%), *E. coli* (n = 0; 0%), and *Enterobacter* spp. (n = 3; 12%). All identified bacteria at enrollment and d 14 were pure growth (no mixed bacteria) at levels ≥100 cfu/mL. Descriptive statistics of the identified bacteria are presented in Table 1. None of the population characteristics differed between the CON and PEG groups (parity: *P* = 0.62; DIM: *P* = 0.83; lactation stage: *P* = 0.68; body temperature at enrollment: *P* = 0.77; proportion of bacteria in milk at enrollment: *P* = 0.77). Nineteen cows (25%) died in the first week post-treatment (16 had *Klebsiella* spp.; 3 had *E. coli*) and 5 (6%) more died or were culled between 7 and 30 d after enrollment (3 had *Klebsiella* spp.; 2 had *E. coli*). The probability of surviving during the first 30 d following treatment was higher in the PEG group than in the control group [PEG: 34/38; 89%, CON: 19/39; 49%; odds ratio (OR)_PEG:CON_ = 5.3; *P* < 0.01; Figure 1]. Of cows that survived until d 14 after enrollment, there was no difference in the proportion of bacteriological cure at d 14 between the PEG and control groups (PEG: 25/36; 69%, CON: 10/22; 45%; OR_PEG:CON_ = 2.4; *P* = 0.12). Similarly, the daily milk production of the cows that survived (n = 53) did not differ between the control and PEG groups over the 30-d period following enrollment (*P*_treatment_ = 0.72; *P*_treatment×day_ = 0.99; Figure 2).

**Table 1:** Descriptive statistics of bacteriology results (cfu/mL) at enrollment and d 14 from 77 cows with severe clinical mastitis enrolled in a randomized double-blinded controlled trial, treated either with pegbovigrastim (PEG) as an adjunct therapy or with saline (CON)

Bacteriology	Statistic^1^	Enrollment (n = 77)		Day 14 (n = 58)	
		PEG (n = 38)	CON (n = 39)	PEG (n = 36)	CON (n = 22)
*Klebsiella* spp. n_enrollment_ = 48 n_d14_ = 22	Minimum	900	700	100	500
	Q1	1,500	1,200	1,200	1,900
	Median	5,000	5,000	1,500	2,800
	Q3	5,000	5,000	2,200	3,000
	Maximum	5,000	5,000	3,000	5,000
*Escherichia coli* n_enrollment_ = 16 n_d14_ = 0	Minimum	800	1,000	NA^2^	NA
	Q1	2,000	2,200		
	Median	5,000	5,000		
	Q3	5,000	5,000		
	Maximum	5,000	5,000		
*Enterobacter* spp. n_enrollment_ = 10 n_d14_ = 3	Minimum	1,100	1,300	700	1,200
	Q1	1,800	1,600	700	1,200
	Median	4,000	4,500	700	1,200
	Q3	5,000	5,000	700	1,700
	Maximum	5,000	5,000	700	1,700

1 Q1, Q3 = first and third quartiles, respectively.

2 Not applicable.

**Figure 1:**
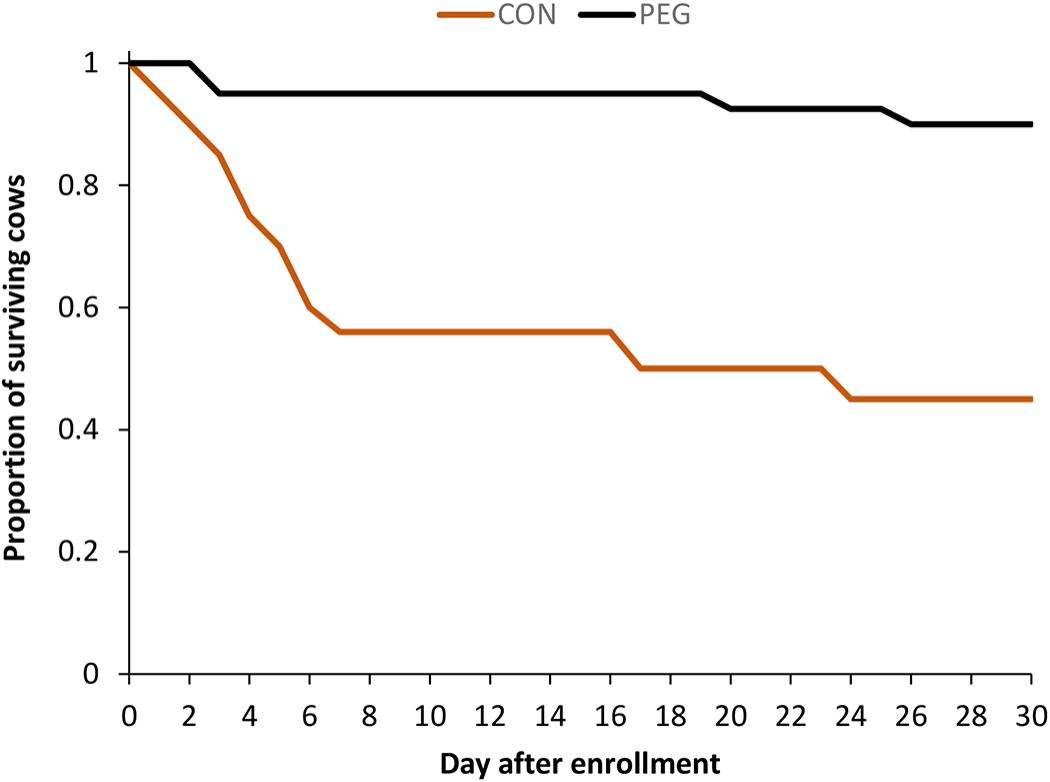
Survival (±95% CI) plotted using Kaplan-Meier estimator of 77 cows with severe clinical mastitis enrolled in a randomized double-blinded controlled trial, treated either with pegbovigrastim (PEG; n = 38; black line) as an adjunct therapy or with saline (CON; n = 39; gray line). Nonsurvival was when cows died or were culled.

**Figure 2:**
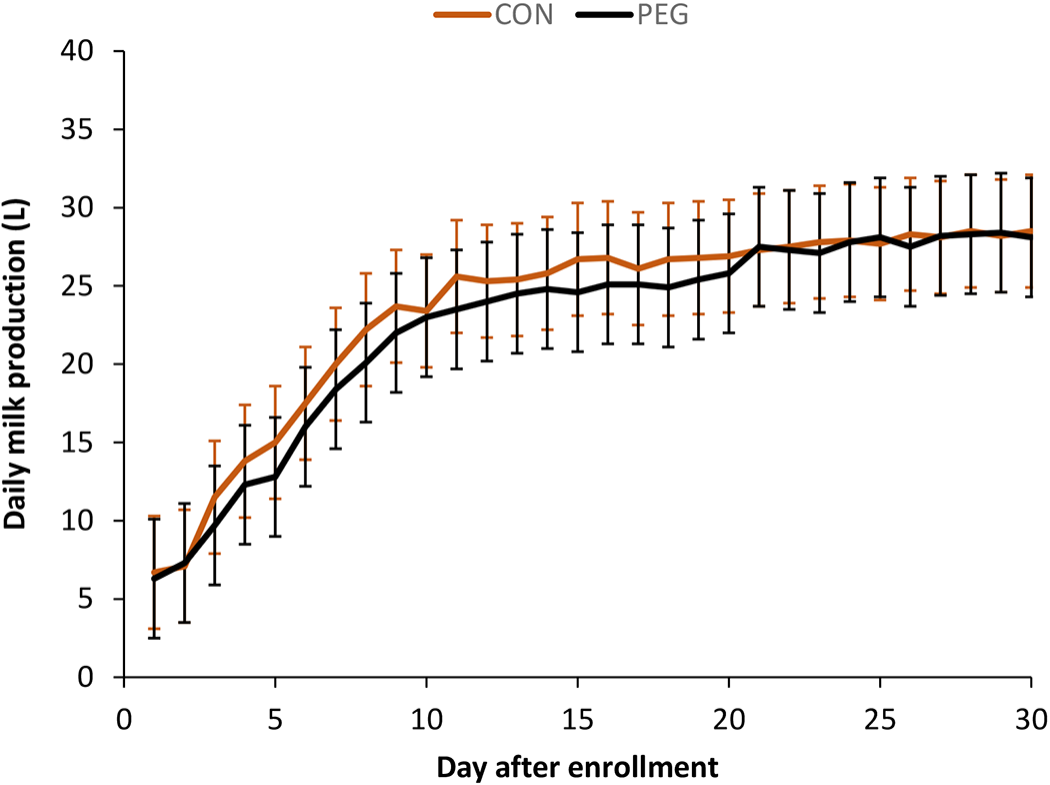
Daily milk production (marginal means ± 95% CI) of 58 cows with severe clinical mastitis enrolled in a randomized double-blinded controlled trial, treated either with pegbovigrastim (PEG; n = 36) or with saline (CON; n = 22) as an adjunct therapy. The marginal means were obtained from a mixed linear regression model using Satterthwaite's method ANOVA for repeated measures and adjusted for DIM as a fixed effect.

Although many studies have looked at the use of PEG for the prevention of mastitis (Hassfurther et al., 2015; Canning et al., 2017; Ruiz et al., 2017) or the treatment of chronic mastitis (Putz et al., 2019), much remains unknown about its use as an adjunct therapy for the treatment of bovine diseases such as naturally occurring severe clinical mastitis (Kehrli et al., 1991). The use of PEG in the present study resulted in better odds of survival during the 30 d following the mastitis event compared with saline (control group). There were, however, no differences in the odds of bacteriological cure on d 14 post-treatment or in daily milk production over the 30-d period after treatment.

The cows enrolled in the present study were all from one farm, and the bacteria identified in milk at mastitis diagnosis were all coliforms, mainly *Klebsiella* spp., with 3 cultures being negative. This is line with the fact that naturally occurring severe clinical mastitis is often caused by coliforms, which may cause a release of LPS endotoxins that results in an acute phase response (Ruegg, 2012). The present results suggest that inferences from the current study might be limited to a population of severe clinical mastitis cases caused by coliforms. Moreover, the high proportion of *Klebsiella* found in this study differs from that in other studies (Gröhn et al., 2004; Ruegg, 2012), suggesting that variability among farms likely limits the external validity of the present results. With an overall proportion of clinical mastitis cases caused by coliforms reported to be between 12 and 40% (Ruegg, 2012), it would be interesting to assess whether the use of PEG would also improve the short-term prognosis (survival), and perhaps other survival and health parameters when other pathogens are involved.

The current results show that when surviving, cows treated with PEG as an adjunct therapy did not have increased odds of bacteriological cure at d 14. It is unclear whether the numerical difference in bacteriological cure observed between the PEG and control groups was not statistically significant due to the absence of association or to the limited sample size (lack of statistical power). Based on the difference in bacteriological cure found in the present study, we would have needed 79 cows per treatment group to show a significant statistical difference. Unfortunately, it was not possible to enroll such a large number of cows in the context of the current study. It is also possible that the single milk sample taken to assess bacteriological cure was insufficient and resulted in misclassification bias (Dohoo et al., 2011). It is important to keep in mind that bacteriological cure in cases of severe clinical mastitis caused by coliforms is generally high (Lago et al., 2011) unless the IMI becomes chronic, which can be the case with *Klebsiella* spp. Future studies could use a parallel interpretation of duplicate or consecutive day samples, which has been shown to improve the sensitivity of bacterial culture for CNS and *Streptococcus* spp. (Dohoo et al., 2011). It is unclear, however, how it would affect the identification of gram-negative pathogens following a severe mastitis case.

As reported in other studies, the preventive use of PEG in the prepartum period did not result in improved subsequent milk production or decreased culling rates over the whole lactation (Kimura et al., 2014; Hassfurther et al., 2015; Ruiz et al., 2017). As the follow-up period in the present study was only 30 d, it is unclear how milk production and survival would have evolved for cows in each treatment group. For example, milk production has been shown to be reduced for up to 70 d following *Klebsiella* and *E. coli* mastitis (Gröhn et al., 2004). Although the present findings suggest a limited long-term effect of using PEG as an adjunct therapy for severe clinical mastitis, more studies are necessary to confirm this. Future studies should also consider collecting SCC data.

The present study found that the use of PEG as an adjunct therapy in cases of naturally occurring severe clinical mastitis resulted in better survival (30 d after enrollment). No effects of treatment on bacteriological cure 14 d post-treatment or on daily milk production during the 30-d period following enrollment were found. These findings should be considered by veterinarians and dairy producers wanting to improve the short-term survival of cows with severe clinical mastitis.
